# Future Tibetan grasslands will decrease: A novel insight from constructive grass species

**DOI:** 10.1016/j.isci.2025.114228

**Published:** 2025-11-26

**Authors:** Guoyong Tang, Qingwan Li, Shunbin Wang, Jinkai Gu, Qinglin Li, Shengjian Xiang, Wanchi Li

**Affiliations:** 1 Chuxiong Normal University, Chuxiong, China; 2 Institute of Highland Forest Science, Chinese Academy of Forestry Sciences, Kunming, China; 3 Institute of International Rivers and Eco-security, Yunnan University, Kunming, China; 4 Yuanmou Desert Ecosystem Research Station, National Long-Term Scientific Research Base of Comprehensive Control, Chuxiong, Yunnan, China

**Keywords:** Physical geography, Environmental science, Plant ecology

## Abstract

Tibet’s grasslands, the region’s dominant ecosystem, are vital for pastoral livelihoods and wildlife habitats. However, their future distribution under combined climate change and anthropogenic pressures remains poorly understood. This study presents an innovative species-level modeling framework to project Tibetan grassland dynamics through the simulated future spatial distribution of 44 constructive grass species. Our results show a net decline of 4.6% in grassland area by 2060, characterized by a 6.7% reduction in productive grasslands yet a 6.6% expansion of wildlife-habitat grasslands. Projected losses are greater under 2°C/century or 5°C/century warming than under intermediate warming trajectories (3°C–4.1°C/century). These findings suggest climate change will challenge pastoral productivity while benefiting Tibetan wildlife, offering key insights for navigating the complex trade-off between sustaining pastoralism and preserving biodiversity in high-altitude grassland ecosystems.

## Highlights

• Future warming will reduce Tibetan grasslands by 4.6% by 2060
• Productive grasslands decline (−6.7%), wildlife habitats expand (+6.7%)
• Modeling 44 constructive species reveals diverse grassland shifts
• Climate-driven grassland loss threatens ecosystem and pastoral stability

## Graphical abstract

**Figure:**
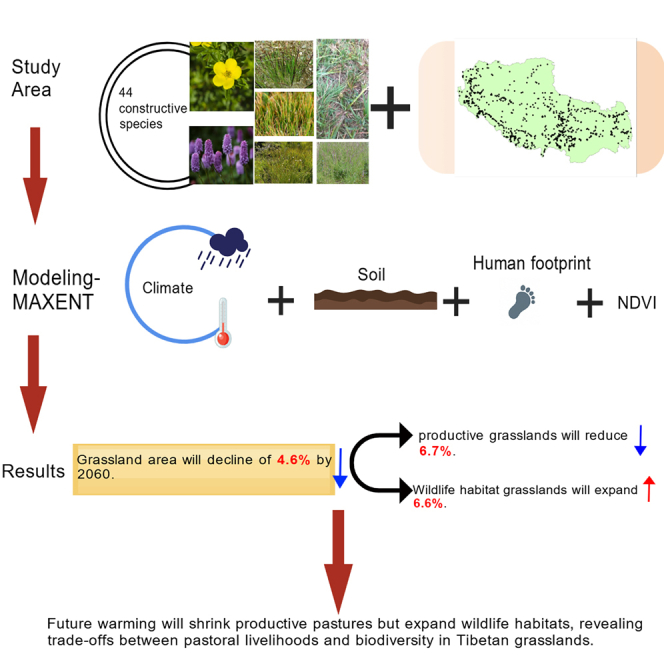

## Introduction

Grasslands dominate Tibet’s landscape, covering ∼75% of its terrain—equivalent to one-sixth of China’s total grassland area.^1^ These ecosystems deliver indispensable services: sustaining pastoral livelihoods through grazing^2^ and harboring endemic wildlife, including Tibetan antelope (*Pantholops hodgsoni*), snow leopard (*Panthera uncia*), and Tibetan wild ass (*Equus kiang*).^3^ Wildlife habitats cluster in the arid, remote alpine desert grasslands of the northwest,^4^ while most Tibetan grasslands are productive, underpinning grazing activities and pastoral livelihoods.^5^

As a globally recognized climate sentinel,^6^ the Tibetan Plateau exhibits complex grassland dynamics.^7^ Satellite observations reveal pervasive greening since the 1980s,^8^ with net primary productivity (NPP) increases across >80% of grasslands (1980–2015), concentrated centrally. Conversely, northwestern sectors degraded under climatic shifts and anthropogenic forcing.^9^ Early gains (1982–2000) were climate-dominated, but post-2000 stagnation reflects intensifying pressures from grazing and land-use change.^10^

Projections of future grassland trajectories diverge markedly. Some studies suggest climate warming could expand grasslands,^11^^,^^12^^,^^13^ citing northwestward transitions from barren land^12^ and modeled vegetation cover increases under high-emission scenarios (e.g., SSP585).^13^ Conversely, others predict elevational contraction of productive grasslands through forest encroachment,^14^^,^^15^^,^^16^^,^^17^ exemplified by high-altitude diminution and downslope forest advancement.^15^ These divergences reflect uncertainties in future trajectories, posing critical challenges for conservation and land-use planning. They stem from methodological and ecological factors: varying spatial scales (coarse regional vs. fine local models), inconsistent grassland classification systems, and overreliance on broad vegetation classes or individual species (neglecting dominant species assemblages that define functional grassland communities). This underscores the need for ecologically grounded, spatially explicit modeling approaches—addressed here by focusing on constructive species.

**Figure 1:**
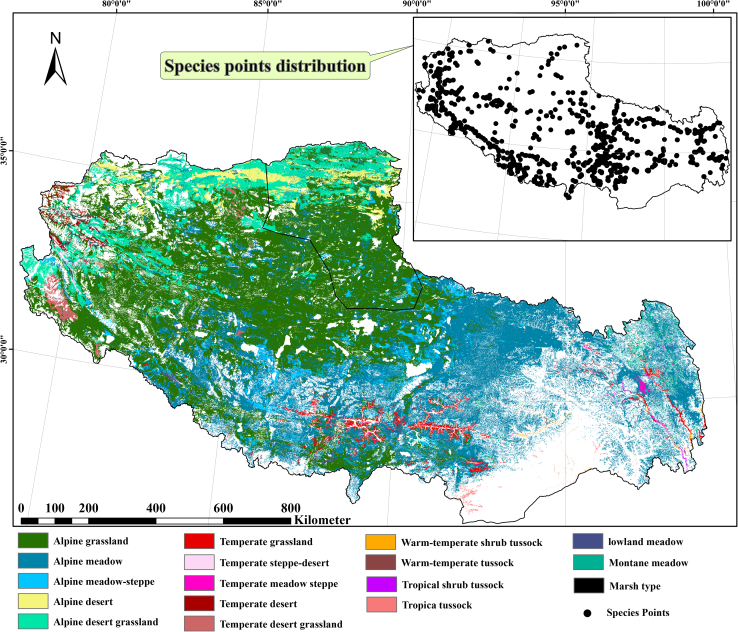
Grassland types and occurrence records of constructive species on Tibet Map showing the distribution of 17 grassland vegetation types across the Tibetan Plateau, with 2,260 occurrence records of 44 constructive grass species overlaid as points. Scale bar, 800 km.

Constructive species—ecological “architects”—shape plant community assembly, stability, and biome identity.^18^^,^^19^ In Tibetan grasslands, dominant constructive species (e.g., *Stipa purpurea*, *Carex moorcroftii*, and *Kobresia pygmaea*) mediate ecosystem processes and biodiversity,^20^ serving as diagnostic indicators of grassland integrity.^21^ This study models distributions of 44 such species representing all 17 Tibetan grassland types (Figure 1). This species-vegetation mapping framework enables nuanced, ecologically robust assessment of future distribution. By focusing on constructive species (rather than single taxa or broad classes), it captures dominant community structure, function, and adaptive responses with greater realism—providing a foundation for understanding ecosystem resilience and informing conservation and land-use strategies under climate change.

Prevailing approaches^22^^,^^23^^,^^24^^,^^25^—ecosystem models (constrained spatial heterogeneity^22^), species distribution models (SDMs) (community-limited^25^), remote sensing (functionally indistinct^25^), or climate–vegetation projections (neglecting local gradients^23^^,^^24^)—fail to integrate ecological dominance with environmental drivers. We therefore (1) develop a constructive species-based framework for grassland dynamics and (2) project 2060 distributions of Tibetan grassland to guide climate-adaptive stewardship. Projections terminate at 2060 due to indeterminacy in post-2060 policy regimes.

## Results

### Distribution of Tibetan grasslands

**Figure 2:**
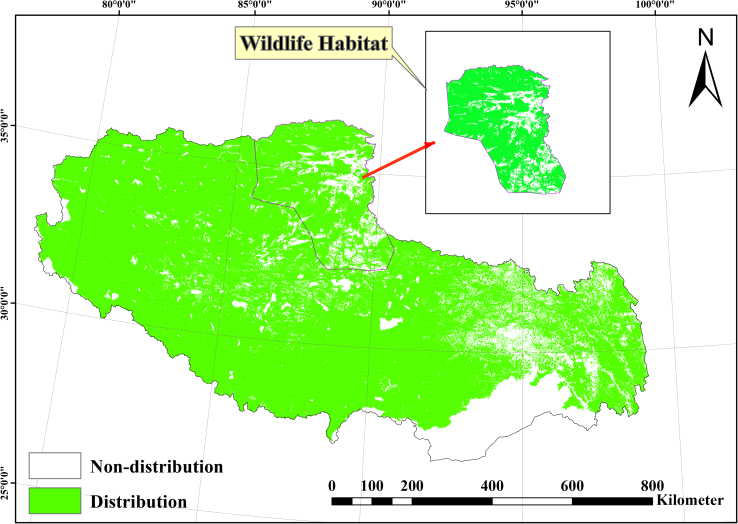
Baseline (2024) Tibetan grassland distribution derived from aggregated habitat suitability of 44 constructive grass species Modeled grassland extent aggregated from MaxEnt predictions of constructive species under baseline climate. Scale bar, 800 km.

The total modeled grassland area in Tibet is approximately 80.03 × 10^4^ km^2^, accounting for 66.54% of the region’s land area (Table 1; Figure 2). This estimate closely aligns with the 71.8% coverage documented in the authoritative second Tibetan grassland survey. The slight discrepancy may be attributed to three factors: temporal variations in data acquisition, grassland boundary delineation uncertainties within ecological transitional zones, and methodological distinctions—notably our exclusion of shrub-grasslands and forest gaps classified as grasslands in the second Tibetan grassland survey.

**Table 1:** Baseline (2024) and projected (2060) Tibetan grassland area under climate scenarios

Climate scenarios	Area(10^4^ km^2^)	Percentage of Tibet (%)	Compare to 2024 (%)
Baseline (2024)	80.03	66.54	–
2060-SSP126	75.72	62.96	−5.39
2060-SSP245	77.78	64.66	−2.82
2060-SSP370	79.69	66.25	−0.43
2060-SSP585	72.22	60.05	−9.76
2060-Mean	76.35	63.48	−4.60

2060-SSP126, 2060-SSP245, 2060-SSP370, 2060-SSP585 denote the projected distributions under 2°C, 3°C, 4.1°C, and 5°C warming scenarios for 2060, respectively; 2060-Mean is the mean distribution across these four scenarios.

Productive grasslands constitute the predominant category, occupying 69.31 × 10^4^ km^2^ (86.36% of total grassland extent), while wildlife-habitat grasslands cover 10.94 × 10^4^ km^2^ (13.64%; Figure 2).

**Figure 3:**
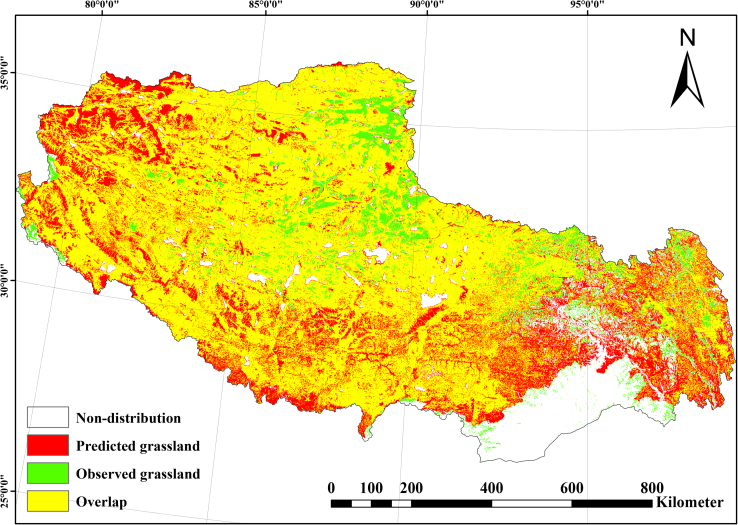
Spatial validation of modeled 2024 grassland distribution against the second Tibetan grassland survey dataset Yellow areas: concordant grasslands (model-survey overlap). Red areas: commission errors (mode-predicted grasslands absent from survey). Green areas: omission errors (survey grasslands absent from model). White areas: consistent non-grasslands. Scale bar, 800 km.

Spatial validation against field survey data from the authoritative second Tibetan grassland survey demonstrated **90.34%** concordance between modeled and observed grassland distributions (Figure 3), confirming high predictive fidelity.

### Projected grassland changes under different climate scenarios

Under multi-scenario projections, Tibetan grasslands are modeled to undergo a net reduction of 4.6% by 2060, with total area declining to an average of 76.35 × 10^4^ km^2^ (63.48% of regional land area) across four climate pathways (Table 1), compared to baseline (2024).

**Figure 4:**
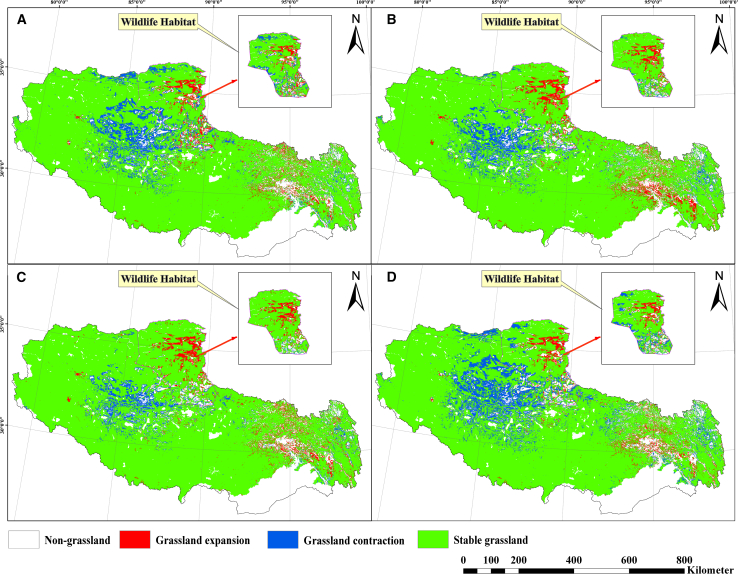
Non-linear responses of Tibetan grassland distribution for 2060 under four climate scenarios relative to baseline (2024) Projected grassland extent under four SSP scenarios: (A) SSP126 (+2°C), (B) SSP245 (+3°C), (C) SSP370 (+4.1°C), and (D) SSP585 (+5°C). Each panel includes a scale bar, 800 km.

Substantial heterogeneity is observed among the four climate scenarios, as illustrated in Figure 4. SSP126 (+2°C) projects grassland contraction to 76.35 × 10^4^ km^2^, corresponding to a 5.39% decrease from 2024 levels. SSP245 (+3°C) yields comparatively milder impacts, reducing grassland cover to 77.78 × 10^4^ km^2^ (−2.82%). SSP370 (+4.1°C) lead to only marginal loss (0.43%), maintaining 79.69 × 10^4^ km^2^ coverage. Conversely, SSP585 (+5°C) causes the most severe degradation, diminishing grasslands to 72.22 × 10^4^ km^2^ (60.05% land share; −9.76%), showed in Table 1 and Figure 4.

### Changes in productive and wildlife-habitat grasslands under climate scenarios

Tibetan productive grasslands exhibit spatially divergent trajectories under the four climate scenarios (SSP126, SSP245, SSP370, and SSP585), expanding in southeastern Tibet while contracting in central, western, and northwestern sectors (Figures 2 and 4). This pattern results in net reductions of −2.93% (SSP370) to −11.58% (SSP585), with pronounced northwest shrinkage under SSP126 and SSP585, highlighting substantial regional heterogeneity.

**Figure 5:**
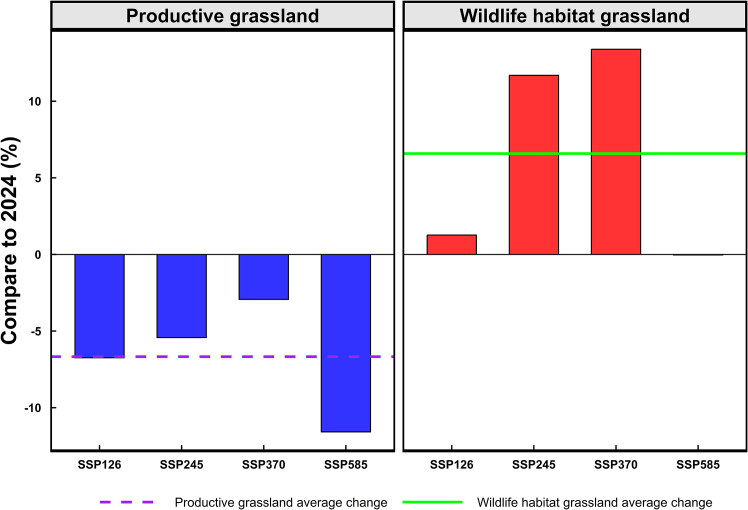
Differential responses of Tibetan grassland functional types to 2060 climate scenarios relative to 2024 baseline Blue bars: changes in productive grasslands. Red bars: changes in wildlife-habitat grasslands. Solid line: mean trajectory of wildlife-habitat grassland. Dashed line: mean trajectory of productive grassland.

Conversely, wildlife-habitat grasslands demonstrate net expansion across all scenarios (+1.27% to +13.39%), compared to 2024. Maximum expansions occur SSP370 (+13.39%), driven predominantly by central region colonization. SSP585 yields marginal net change (−0.03%) due to southern contractions offsetting central expansions. These patterns establish the central region as pivotal for future wildlife-habitat distribution (Figure 5).

## Discussion

### Future changes in Tibetan grasslands

Our multi-scenario projections consistently indicate net grassland contraction across the Tibetan Plateau, reinforcing prior evidence of Tibetan grassland’s vulnerability to climate warming.^14^^,^^15^^,^^26^^,^^27^ Critically, the Tibetan Plateau is warming at 1.3–1.45 times the global average,^26^ which amplifies vegetation redistribution and drives substantial grassland contraction, particularly at high altitudes. Thus, our findings, like those of others,^14^^,^^15^^,^^16^ underscore that amplified warming acts as a primary catalyst for high-altitude grassland loss.

The projected reduction in grassland area across all scenarios is likely driven by rising temperatures.^28^ Elevated temperatures induce soil aridification, exacerbate water stress, and thereby limit vegetation growth and accelerate grassland degradation. Ji et al.^27^ reported that increased evaporation rates driven by higher temperatures significantly deplete soil moisture, rendering grassland ecosystems high vulnerability. Although certain scenarios project modest precipitation increases, these remain insufficient to counterbalance warming-induced water deficits given persistent negative water balance, which are inadequate to support grassland expansion.^29^

Interestingly, grassland responses exhibit non-linear sensitivity to warming intensity. The marginal 0.7% contraction under SSP 370 (+4.1°C) contrasts sharply with substantial losses under SSP126 (−5.6%) and SSP585 (−10%). This suggests that precipitation variability may exert compensatory effects at moderate warming scenarios like SSP245 and SSP370, partially offsetting thermal stress^27^ through soil moisture recharge—though such buffering remains spatially and scenario-dependent.

Hydrothermal interactions further modulate the decrease of Tibetan grassland. NDVI and climate data show that over 60% of Tibet’s vegetated areas demonstrate precipitation-limited growth dynamics under warming scenarios.^30^ Cold-arid regions (MAT <10 °C; MAP <650 mm) experience pronounced productivity inhibition under elevated vapor pressure deficit (VPD). Conversely, warm-humid zones may benefit from analogous conditions.^31^ These divergent patterns underscore the necessity of incorporating regional water-energy equilibria when interpreting non-linear grassland responses to climate changes, particularly under intermediate warming scenarios like SSP370.^32^

### Future changes in productive and wildlife-habitat grasslands

Productive grasslands are projected to decline substantially under future climates, particularly under extreme warming conditions, such as SSP585 (+5°C). This trend concurs with other works^14^^,^^33^ emphasizing the vulnerability of Tibetan grasslands to climate warming. Huang et al.^33^ empirically documented reductions in net ecosystem productivity across western Tibet, indicative of fundamental vegetation degradation. These contractions in productive grasslands under high-temperature scenarios directly threaten pastoral sustainability by diminishing grazing resources, potentially accelerating edaphic nutrient depletion and biodiversity erosion.^31^^,^^34^ Consequently, implementing strict stocking controls emerges as an essential conservation imperative.

Conversely, wildlife-habitat grasslands demonstrate net expansion, especially in northwestern Tibet under future climate scenarios. Three primary drivers underpin this trend: First, climatic amelioration through modest precipitation increases and warming enhances habitat suitability in the historically harsh, precipitation-limited alpine desert grasslands of the northwest.^13^^,^^27^ Second, cryospheric retreat and vegetation migration generate newly viable territories for wildlife habitats. Third, anthropogenic disintensification via state-led herder relocation to lower-altitudes reduces landscape pressure.^35^ This transformation converts degraded pastures into refugia for cryophilic species, though such areal gains constitute ecological reallocation rather than qualitative enhancement.

Critically, this redistribution coincides with deterioration of core ecosystem services. Rising temperatures and precipitation volatility in transition zones may compromise soil nutrient cycling and carbon sequestration capacity.^36^ Moreover, the functional conversion from productive grasslands to wildlife habitats represents a net loss in provisioning services despite superficial expansion.^37^ This superficial expansion of wildlife-habitat grasslands may involve ecological trade-offs. Vegetation community restructuring further diminishes soil organic carbon stocks and NPP, particularly where shrubs or sparse vegetation encroach replace herbaceous assemblages in high-altitude or marginal areas.^38^ Permafrost degradation compounds these issues by mobilizing historically stable carbon reservoirs, fundamentally undermining regional carbon sink resilience despite elevated nitrogen mineralization.^39^

These findings necessitate a paradigm shift in conservation strategy. Future land management must transcend habitat-centric metrics to holistically evaluate functional trade-offs, particularly regarding carbon-climate feedback in thermally sensitive alpine ecosystems. Policy interventions should prioritize maintaining critical ecosystem processes alongside biodiversity conservation, especially under moderate warming scenarios where compensatory mechanisms may remain operative.

### Modeling Tibetan grasslands via constructive species: Applications and limitations

This study advances grassland distribution modeling by employing SDMs incorporating 44 constructive grass species representing dominant Tibetan vegetation types. By integrating multiple ecologically representative species, our framework provides a more robust assessment of habitat suitability under climate change than single-indicator approaches. SDMs’ capacity to synthesize diverse environmental predictors (e.g., climate, edaphic factors, vegetation indices, and topography) enhances their applicability for projecting ecosystem responses to climatic shifts.^40^

Several limitations warrant acknowledgment. While SDMs effectively capture abiotic drivers, they neglect critical biotic interactions (e.g., interspecific competition/facilitation) that shape community assembly. Our pixel-level aggregation method selects maximum suitability values across species, which may overestimate ecological trade-offs and habitat continuity. Furthermore, the static species-environment relationships fail to account for dynamic processes like permafrost degradation, vegetation succession, and anthropogenic disturbance.^41^^,^^42^^,^^43^ These limitations necessitate refined model parameterization and integration of temporal dynamics.

Future research should prioritize three enhancements: First, parameter optimization via tools like ENMeval (particularly for focal species studies), overcoming the standardized MaxEnt settings applied here due to computational constraints. Second, integrating high-resolution spatial datasets (e.g., 250-m land cover), dynamic variables (land-use change, permafrost thaw), and empirical co-occurrence data to strengthen ecological validity. Third, promoting open data practices to enable reproducibility and cross-model validation.

### Limitations of the study

This study has several limitations. First, while SDMs capture abiotic drivers of grassland distribution, they neglect biotic interactions, such as competition and facilitation, which may influence species assembly. Second, our pixel-level aggregation approach, which selects maximum suitability values, may overestimate habitat continuity and ecological trade-offs. Third, the static species-environment relationships used here do not incorporate dynamic processes, such as permafrost degradation, vegetation succession, or anthropogenic disturbance. Finally, although MaxEnt provided robust predictions across 44 constructive species, parameter optimization (e.g., ENMeval) and integration of higher-resolution and dynamic datasets would further enhance model realism. Future research should address these limitations to strengthen the robustness of Tibetan grassland projections.

## Resource availability

### Lead contact

Requests for further information and resources should be directed to and will be fulfilled by the lead contact, Guoyong Tang (tangguoyong1980@caf.ac.cn).

### Materials availability

This study did not generate new unique reagents.

### Data and code availability

• All environmental datasets and species occurrence records used in this study, including primary data for the 44 constructive grass species across the Tibetan Plateau deposited in Figshare (https://doi.org/10.6084/m9.figshare.30290848), are publicly accessible from the sources listed in the key resources table.
• Model outputs, processed distribution maps, and analysis scripts supporting the findings of this study are available from the lead contact upon reasonable request.
• This paper does not report original code. Species distribution modeling was conducted using the MaxEnt v3.4.4 platform (publicly available at https://biodiversityinformatics.amnh.org/open_source/maxent/).
• Any additional information required to reanalyze the data reported in this paper is available from the lead contact upon request.

## Acknowledgments

This research was funded by the 10.13039/501100001809National Natural Science Foundation of China (no. 32471714).

## Author contributions

Q.W.L. (Li) and G.Y.T. (Tang) conceived and designed the project; Q.W.L. (Li) collected the data; Q.W.L. (Li), S.B.W. (Wang), J.K.G. (Gu), Q.L.L. (Li), S.J.X. (Xiang), W.C.L. (Li), and G.Y.T. (Tang) conducted the analysis and contributed to writing the manuscript; Q.W.L. (Li) and G.Y.T. (Tang) participated in revising the manuscript; all authors have read and approved the final version.

## Declaration of interests

The authors declare no competing interests.

## STAR★Methods

### Key resources table

**Table:** 

REAGENT or RESOURCE	SOURCE	IDENTIFIER
**Antibodies**		

Species occurrence records	Global Biodiversity Information Facility (GBIF)	https://www.gbif.org
Species occurrence records	National Specimen Resources Sharing Platform (NSII)	http://www.naii.org.cn
Species occurrence records	Chinese Virtual Herbarium (CVH)	http://www.cvh.ac.cn

**Deposited data**		

Climate data (current & future)	WorldClim v2.1	https://www.worldclim.org
Future climate scenarios (BCC-CSM2-MR, SSP126/245/370/585)	CMIP6 archive, WorldClim v2.1	https://www.worldclim.org
Species distribution modeling outputs for 44 constructive grass species	Figshare	https://doi.org/10.6084/m9.figshare.30290848
Soil data	FAO Harmonized World Soil Database	http://www.fao.org/soils-portal
Vegetation index (NDVI)	Resource and Environmental Science and Data Center (RESDC), China	http://www.resdc.cn
Human footprint index	Center for International Earth Science Information Network (CIESIN)	https://sedac.ciesin.columbia.edu

**Software and algorithms**		

Species distribution modeling software	MaxEnt v3.4.4	https://biodiversityinformatics.amnh.org/open_source/maxent
Statistical and spatial analysis software	R software v4.3.4	https://www.r-project.org
GIS software	ArcGIS v10.8	https://www.esri.com
R packages: biomod2, ggplot2, dplyr, terra	CRAN	https://cran.r-project.org

### Experimental model and study participant details

This study did not involve experimental models, human participants, animals, plants, cell lines, or microbial strains. All analyses were conducted using publicly available species occurrence records and environmental datasets. No experimental manipulation or *in vivo*/*in vitro* systems were used.

### Method details

For enhanced methodological transparency, our workflow included: (1) collecting species records and environmental variables, (2) preprocessing data and screening variables, (3) modeling species distributions using MaxEnt, (4) aggregating habitats to generate composite grassland maps, (5) validating the models with survey data, (6) detecting changes in gains and losses, and (7) interpreting climate impacts.

#### Study area

The Tibet Plateau (78°25′-99°06′E, 26°44′-36°32′N; ∼4,000 m mean elevation) features high-altitude grasslands covering ∼882,015 km^2^. Climate gradients exhibit mean annual temperature of -2.8°C to 11.9°C and precipitation decreasing southeast-northwest (360-550 mm/yr). Grasslands comprise productive (*∼*771,659 km^2^) and wildlife-habitat (*∼*110,356 km^2^) types (unpublished governmental data).

To illustrate the representativeness of the selected species and validation sites, we provide a map of the 17 vegetation types and the spatial distribution of the 44 constructive species points across the Tibetan Plateau (Figure 1).

#### Constructive species selection

Forty-four constructive grass species representing all 17 Tibetan grassland types were selected based on: (1) quantitative dominance (>5% relative cover within host vegetation types, (2) ecological significance, according to the Second Tibetan Grassland Survey, regional vegetation monographs, and peer-reviewed literature. A full species list with corresponding vegetation types is in Table S1.

Focusing on these dominant constructive species ensures our constructive species-based framework captures core ecological composition and landscape representativeness, enabling ecologically grounded assessments of vegetation distribution and future shifts.

In total, 2260 occurrence points were compiled for the 44 species, covering all 17 vegetation types across the Tibetan Plateau (Figure 1). Most vegetation types were well represented, whereas a few minor types had limited records due to their naturally restricted extent and low dominance.

#### Modeling framework

Maximum Entropy models (MaxEnt v3.4.4)^40^^,^^43^^,^^44^ were used to project current and 2060 distributions of the 44 constructive species under future climate scenarios. Key variables (climate, human footprint, topography, soil, vegetation, and aridity index) are major drivers of Tibetan grassland ecosystems,^45^ providing a comprehensive basis for assessing distribution under environmental change. Detailed environmental variables and modeling parameters are summarized in Table S2.

#### Data processing

The 44 constructive grass species, identified from the Second Tibetan Grassland Survey, represent all major grassland types across the Tibetan Plateau. Species occurrence data (1970–2024) were compiled from the Global Biodiversity Information Facility (http://www.gbif.org), the National Specimen Resource Sharing Platform (http://www.naii.org.cn), and the Chinese Virtual Herbarium (http://www.cvh.ac.cn). Records were filtered for data quality and spatial independence using EnmTools, retaining one occurrence per 1 km × 1 km grid cell.

Current climate data (1970–2020 average) and 2060 projections were obtained from WorldClim v2.1 (http://www.worldclim.org). Future projections were based on the CMIP6 BCC-CSM2-MR model (optimized for China) under four Shared Socioeconomic Pathways (SSPs): SSP126 (sustainable development, +2°C), SSP245 (moderate development, +3°C), SSP370 (regional rivalry, +4.1°C), and SSP585 (fossil-fueled development, +5°C).

Additional variables included soil data (pH, bulk density, organic carbon) from FAO’s Harmonized World Soil Database (http://www.fao.org/soils-portal), the Normalized Difference Vegetation Index (NDVI) from the Resource and Environmental Science and Data Center of China (http://www.resdc.cn), and the Human Footprint Index from the Center for International Earth Science Information Network (CIESIN; http://ciesin.org), which integrates population density, infrastructure, and land-use intensity.

The 44 constructive species representing distinct Tibetan grassland formations (Table S1) were used as ecological proxies for modeling grassland dynamics. Environmental predictors (Table S2) encompassed bioclimatic indices, soil properties, topographic metrics, drought indices, vegetation indices (NDVI), and anthropogenic pressure indicators. All environmental layers were resampled to 30 arc-second (∼1 km) resolution.

#### Model calibration

MaxEnt v3.4.4 simulated current and 2060 distributions of the 44 constructive grass species. Models were calibrated with 75% of occurrence data for training and 25% for testing, using 10-replicate cross-validation. Regularization multiplier was set to 2 to reduce overfitting; feature types (linear, hinge, product) were auto-selected by sample size. Given the number of species modeled (*n* = 44), ENMeval optimization was not used; species-specific variable filtering and cross-validation ensured robustness.

Multicollinearity was reduced through an iterative variable filtering procedure implemented within MaxEnt to minimize overfitting among environmental predictors. For each species, preliminary simulations included all environmental variables, and variables showing zero contribution were systematically excluded. Pairwise correlation analysis (|r| ≥ 0.8^46^) was then applied, retaining only the variable with higher contribution from each correlated pair. This species-specific screening ensured ecological interpretability of environmental gradients. Static parameters such as soil properties were assumed temporally invariant and maintained current values in future projections.

Model performance was evaluated via the Area Under the Receiver Operating Characteristic Curve (AUC); all species models had AUC > 0.9 (high reliability). AUC values ≤ 0.6 were considered poor, 0.6–0.8 moderate, and ≥ 0.8 high predictive accuracy. All species models exceeded AUC ≥ 0.8, confirming robust predictive performance.

#### Habitat mapping

Continuous MaxEnt suitability outputs (raster, ASC) were thresholded into binary presence–absence maps using the maximum sensitivity–specificity sum criterion. In addition, continuous suitability surfaces (0–1 probability) were reclassified into four discrete tiers: non-suitable (*P* < 0.2), low (0.2 ≤ *P* < 0.4), medium (0.4 ≤ *P* < 0.6), and high suitability (*P* ≥ 0.6). The combined area of low to high suitability tiers was defined as the total potential grassland habitat. Composite grassland distribution maps were then generated by spatially integrating species-specific projections in ArcGIS 10.8.

#### Change detection

Baseline (2024) and projected (2060) grassland distribution maps were converted to vector format. We quantified areal changes using ArcGIS spatial analyst tools (Intersect and Tabulate Area) and visualized results with R ggplot2 / dplyr packages. Species suitability maps were reclassified to binary, aggregated, and net expansion/contraction calculated *via* raster math.

#### Model validation

Modeled 2024 Tibetan grassland distribution showed **90.34%** spatial agreement (strong consistency) with the authoritative Second Tibetan Grassland Survey datasets (Figure 3), assessed using ArcGIS spatial analyst tools (Tabulate Area and Intersect). Filed data confidentiality protocols were maintained. These results support the model framework’s robustness and suitability for future projections.

#### Spatial masking

Non-grassland land uses (e.g., urban areas, infrastructure, permanent snow, permafrost zones) were masked out using official land-use data layers prior to analysis. This step ensured that modeled grassland projections were limited to biologically plausible areas.

### Quantification and statistical analysis

Model performance was assessed using the Area Under the Receiver Operating Characteristic Curve (AUC) derived from 10-fold cross-validation.

Continuous habitat suitability outputs were classified into four tiers: non-suitable (*P* < 0.2), low (0.2 ≤ *P* < 0.4), medium (0.4 ≤ *P* < 0.6), and high (*P* ≥ 0.6).

Baseline (2024) and projected (2060) distribution maps were compared in ArcGIS (Intersect and Tabulate Area) and R (ggplot2, dplyr, terra) to quantify net expansion and contraction areas.
