# Supplementary material for: Future Tibetan grasslands will decrease: A novel insight from constructive grass species
Source: iScience. 2025 Nov 26;28(12):114228. doi: 10.1016/j.isci.2025.114228 (PMC12767175; doi:10.1016/j.isci.2025.114228)
Supplement: Document S1. Tables S1 and S2 [file mmc1.pdf]

iScience, Volume *28*

Supplemental information

Future Tibetan grasslands will decrease: A novel insight from constructive grass species

Guoyong Tang, Qingwan Li, Shunbin Wang, Jinkai Gu, Qinglin Li, Shengjian Xiang, and Wanchi Li

Table S1: Tibetan constructive grass species characterization

Grass Species	Family	Genus	Grassland Type	Distribution Points
Ajania fruticulosa	Asteraceae	*Ajania*	Temperate steppe-desert, Temperate desert	23
Argentina anserina	Rosaceae	*Argentina*	Azonal lowland meadow	67
Artemisia minor	Asteraceae	*Artemisia*	Alpine grassland	70
Artemisia stracheyi	Asteraceae	*Artemisia*	Alpine grassland	61
Artemisia vestita	Asteraceae	*Artemisia*	Temperate grassland	47
Artemisia wellbyi	Asteraceae	*Artemisia*	Temperate grassland, Alpine grassland	77
Artemisia younghusbandii	Asteraceae	*Artemisia*	Temperate grassland, Alpine grassland	22
Bistorta macrophylla	Polygonaceae	*Bistorta*	Montane meadow	102
Bistorta vivipara	Polygonaceae	*Bistorta*	Montane meadow	25
Caragana versicolor	Fabaceae	*Caragana*	Temperate desert grassland, Alpine grassland, Alpine desert grassland	50
Carex alatauensis	Cyperaceae	*Carex*	Montane meadow, Alpine meadow	54
Carex cercostachys	Cyperaceae	*Carex*	Alpine meadow	36
Carex deasyi	Cyperaceae	*Carex*	Alpine meadow	56
Carex littledalei	Cyperaceae	*Carex*	Alpine meadow	53
Carex moorcroftii	Cyperaceae	*Carex*	Alpine meadow, Alpine grassland, Alpine desert grassland	67
Carex neesii	Cyperaceae	*Carex*	Alpine meadow	18
Carex parvula	Cyperaceae	*Carex*	Alpine meadow, Alpine meadow-steppe	144
Carex tibetikobresia	Cyperaceae	*Carex*	Alpine meadow	21

Christolea crassifolia	Brassicaceae	*Christolea*	Alpine desert	39
Dasiphora fruticosa	Rosaceae	*Dasiphora*	Alpine grassland	90
Dasiphora parvifolia	Rosaceae	*Dasiphora*	Alpine grassland	58
Elymus nutans	Poaceae	*Elymus*	Montane meadow	86
Juniperus pingii	Cupressaceae	*Juniperus*	Alpine meadow-steppe	46
Krascheninnikovia ceratoides	Amaranthaceae	*Krascheninnikovia*	Alpine desert grassland, Temperate desert, Temperate steppe-desert	50
Krascheninnikovia compacta	Amaranthaceae	*Krascheninnikovia*	Alpine desert grassland, Alpine desert	16
Leymus secalinus	Poaceae	*Leymus*	Alpine meadow	74
Myrtama elegans	Tamaricaceae	*Myrtama*	Azonal lowland meadow	13
Orinus thoroldii	Poaceae	*Orinus*	Alpine grassland, Temperate steppe-desert	67
Pennisetum flaccidum	Poaceae	*Pennisetum*	Temperate grassland	57
Poa alpina	Poaceae	*Poa*	Alpine meadow	14
Poa litwinowiana	Poaceae	*Poa*	Montane meadow	38
Puccinellia himalaica	Poaceae	*Puccinellia*	Alpine meadow	27
Sophora davidii	Fabaceae	*Sophora*	Warm-temperate shrub tussock	16
Sophora moorcroftiana	Fabaceae	*Sophora*	Temperate grassland	30
Spiraea canescens	Rosaceae	*Spiraea*	Temperate grassland, Montane meadow	43
Stevenia canescens	Brassicaceae	*Stevenia*	Alpine desert, Montane meadow	30

Stipa bungeana	Poaceae	*Stipa*	Temperate grassland	18
Stipa capillacea	Poaceae	*Stipa*	Temperate meadow-steppe, Alpine meadow-steppe	34
Stipa caucasica	Poaceae	*Stipa*	Temperate desert grassland, Temperate steppe-desert, Alpine desert grassland	43
Stipa purpurea	Poaceae	*Stipa*	Alpine grassland, Alpine meadow-steppe	230
Stipa roborowskyi	Poaceae	*Stipa*	Alpine grassland	52
Stipa subsessiliflora	Poaceae	*Stipa*	Alpine grassland	32
Suaeda corniculata	Amaranthaceae	*Suaeda*	Alpine meadow	24
Triglochin palustris	Juncaginaceae	*Triglochin*	Marsh type rangeland	40

Table S2: Data sources and environmental parameterization

Data Source	Dataset	Description and Unit	Available at
Climate	bio1*	Annual Mean Temperature (°C)	http://www.worldclim.org
	bio2*	Mean Diurnal Range (°C)	
	bio3*	Isothermality	
	bio4*	Temperature Seasonality (*)	
	bio5*	Max Temperature of Warmest Month (°C)	
	bio6*	Min Temperature of Coldest Month (°C)	
	bio7*	Annual Temperature Range (°C)	
	bio8*	Mean Temperature of Wettest Quarter (°C)	
	bio9*	Mean Temperature of Driest Quarter (°C)	
	bio10*	Mean Temperature of Warmest Quarter (°C)	
	bio11*	Mean Temperature of Coldest Quarter (°C)	
	bio12*	Annual Precipitation (mm)	

	bio13*	Precipitation of Wettest Month (mm)	
	bio14*	Precipitation of Driest Month (mm)	
	bio15*	Precipitation Seasonality (Coefficient of Variation)	
	bio16*	Precipitation of Wettest Quarter (mm)	
	bio17*	Precipitation of Driest Quarter (mm)	
	bio18*	Precipitation of Warmest Quarter (mm)	
	bio19*	Precipitation of Coldest Quarter (mm)	
Soil factors	t-esp*	Exchangeable sodium percentage (%)	http://www.fao.org/soil-portal
	t-ece*	Soil electrical conductivity (dS/m)	
	t-clay*	Clay content (%wt)	
	t-cec-soil*	Cation exchange capacity (cmol/kg)	
	t-cec-clay*	Soil cation exchange content (cmol/kg)	
	t-CaSO4*	Soil sulfate content (%weight)	
	t-CaCO3*	Soil carbonate content (%weight)	
	t-bs*	Base saturation (%)	
	t-gravel*	Soil gravel content (%vol.)	
	t-oc*	Soil organic carbon content (%weight)	
	t-ph-H2O*	Soil pH (-log(H+))	
	t-ref-bulk*	Soil bulk density (kg/dm³)	
	t-sand*	Sand content (%wt.)	
	t-silt*	Silt content (%wt.)	
	t-teb*	Soil exchangeable base (cmol/kg)	
	t-tusda-tex-clay*	Soil texture classification (name)	
Topographic factors	aspect*	Aspect (°)	https://www.gscloud.cn
	dem	Elevation (m)	
	slope*	Slope (°)	
Drought factors	ai*	Aridity index (%)	http://data.tpdc.ac.cn
Other factors	ndvi*	Normalized difference vegetation index	http://www.resdc.cn
	hfp*	Human footprint	http://ciesin.org

Tibet Plateau	-	-	https://www.gscloud.cn

Environmental variables with an asterisk (*) exhibit 1000-m resolution, while those without an asterisk exhibit 30-m resolution; Edaphic factors with t-prefixed fields denote topsoil characteristics (0–30 cm).
